# Sequencing and curation strategies for identifying candidate glioblastoma treatments

**DOI:** 10.1186/s12920-019-0500-0

**Published:** 2019-04-25

**Authors:** Mayu O. Frank, Takahiko Koyama, Kahn Rhrissorrakrai, Nicolas Robine, Filippo Utro, Anne-Katrin Emde, Bo-Juen Chen, Kanika Arora, Minita Shah, Heather Geiger, Vanessa Felice, Esra Dikoglu, Sadia Rahman, Alice Fang, Vladimir Vacic, Ewa A. Bergmann, Julia L. Moore Vogel, Catherine Reeves, Depinder Khaira, Anthony Calabro, Duyang Kim, Michelle F. Lamendola-Essel, Cecilia Esteves, Phaedra Agius, Christian Stolte, John Boockvar, Alexis Demopoulos, Dimitris G. Placantonakis, John G. Golfinos, Cameron Brennan, Jeffrey Bruce, Andrew B. Lassman, Peter Canoll, Christian Grommes, Mariza Daras, Eli Diamond, Antonio Omuro, Elena Pentsova, Dana E. Orange, Stephen J. Harvey, Jerome B. Posner, Vanessa V. Michelini, Vaidehi Jobanputra, Michael C. Zody, John Kelly, Laxmi Parida, Kazimierz O. Wrzeszczynski, Ajay K. Royyuru, Robert B. Darnell

**Affiliations:** 1 New York Genome Center, 101 Avenue of the Americas, New York, NY 10013 USA; 2 Laboratory of Molecular Neuro-Oncology, The Rockefeller University, 1230 York Avenue, New York, NY 10065 USA; 3 IBM Thomas J. Watson Research Center, Yorktown Heights, NY 10598 USA; 4 Northwell Health, Lenox Hill Hospital, 100 E. 77th Street, New York, NY 10075 USA; 5 Northwell Health, The Brain Tumor Center, 450 Lakeville Road, Lake Success, Lakeville, NY 11042 USA; 6 New York University, School of Medicine, 550 First Avenue, New York, NY 10016 USA; 7 Memorial Sloan-Kettering Cancer Center, 1275 York Avenue, New York, NY 10065 USA; 8 Columbia University Medical Center, 710 West 168th Street, New York, NY 10032 USA; 9 Hospital for Special Surgery, 535 E. 70th Street, New York, NY 10021 USA; 10 IBM Watson Health, NW Broken Sound Bkwy, Boca Raton, FL 33487 USA; 11 Howard Hughes Medical Institute, The Rockefeller University, 1230 York Avenue, New York, NY 10065 USA; 12 Present address: Google, 76 9th Avenue, New York, NY 10011 USA; 13 Present address: Rockefeller University, 1230 York Avenue, New York, NY 10065 USA; 14 Present address: 23&Me, 899 W Evelyn Ave, Mountain View, CA 94041 USA; 15 Present address: Max Planck Institute of Immunobiology and Epigenetics, Stübeweg 51 D-79108, Freiburg, Germany; 16 Present address: The Scripps Research Institute, 10550 N. Torrey Pines Road, La Jolla, CA 92037 USA; 17 Present address: The Tisch Cancer Institute, 1470 Madison Avenue, New York, NY 10029 USA; 18 Present address: Memorial Sloan-Kettering Cancer Center, 1275 York Avenue, New York, NY 10065 USA; 19 Present address: Harvard Medical School, 10 Shattuck Street, Boston, MA 02115 USA; 20 Present address: Yale School of Medicine, 333 Cedar Street, New Haven, CT 06510 USA

## Abstract

**Background:**

Prompted by the revolution in high-throughput sequencing and its potential impact for treating cancer patients, we initiated a clinical research study to compare the ability of different sequencing assays and analysis methods to analyze glioblastoma tumors and generate real-time potential treatment options for physicians.

**Methods:**

A consortium of seven institutions in New York City enrolled 30 patients with glioblastoma and performed tumor whole genome sequencing (WGS) and RNA sequencing (RNA-seq; collectively WGS/RNA-seq); 20 of these patients were also analyzed with independent targeted panel sequencing. We also compared results of expert manual annotations with those from an automated annotation system, Watson Genomic Analysis (WGA), to assess the reliability and time required to identify potentially relevant pharmacologic interventions.

**Results:**

WGS/RNAseq identified more potentially actionable clinical results than targeted panels in 90% of cases, with an average of 16-fold more unique potentially actionable variants identified per individual; 84 clinically actionable calls were made using WGS/RNA-seq that were not identified by panels. Expert annotation and WGA had good agreement on identifying variants [mean sensitivity = 0.71, SD = 0.18 and positive predictive value (PPV) = 0.80, SD = 0.20] and drug targets when the same variants were called (mean sensitivity = 0.74, SD = 0.34 and PPV = 0.79, SD = 0.23) across patients. Clinicians used the information to modify their treatment plan 10% of the time.

**Conclusion:**

These results present the first comprehensive comparison of technical and machine augmented analysis of targeted panel and WGS/RNA-seq to identify potential cancer treatments.

## Background

Oncology is one of the first areas where next-generation sequencing is being applied [1–3]. Sequencing is used to identify genetic variants that could be pharmacologically targeted, allowing identification of drug effects in stratified populations that may otherwise be missed [2, 4]. Panel-based sequencing, using hybridization and capture of specific regions of key genes or of all genes (whole exomes; WES), versus whole genome sequencing (WGS) are different technologies with different costs that have not previously been directly compared. 10,000 cancer patients sequenced with the MSK-IMPACT panel identified potentially clinically actionable calls in 36.7% of individuals sequenced [5]. Deep sequencing coverage increases sensitivity for rare variants in heterogeneous tumors. WGS, however, does not rely on hybridization and capture, a source of potential bias, and is able to identify non-coding variants such as enhancer bindings sites [6] and increases sensitivity for small copy number variants (CNVs) and missense mutations, indels, [7] intronic variants, [8] and gene fusions [9]. The relative impact of of these technologies on making clinically actionable variant calls is unknown.

Panel-based sequencing is used to identify treatment targets in tumors including glioblastoma (GBM) [10–13], the most common adult brain malignancy with a median survival of 14.2 months [14]. The Cancer Genome Atlas (TCGA) analyzed GBM and established four molecular subtypes [15] defined by IDH1 mutation and methylation status, [16] and more recently three subtypes which takes into consideration tumor purity and heterogeneity, [17] but has not yet led to new therapies. Panel sequencing and WGS/RNA-sequencing (WGS/RNA-seq) provide logical paths forward to identify variants. However, the process of sequence analysis and prioritizing variants is laborious, requiring highly trained experts, particularly in WGS/RNA-seq; this prompted prompting assessment of automated analyses.

The New York Glioblastoma Genome Consortium (NY-GGC) was organized in 2013 at the Rockefeller University and New York Genome Center (NYGC) to conduct a feasibility study of using WGS/RNA-seq to identify tumor-specific variants and potential drug targets, to compare WGS/RNA-seq to panels, and to assess the reliability of automated versus manual analyses. Here, we describe an integrated analysis of 30 GBM patients recruited through seven participating institutions.

## Methods

### Study design

The NY-GGC was formed in 2013 through a collaboration initiated at Rockefeller University, and included Memorial Sloan Kettering Cancer Center, New York University Medical School, Northwell Health (Lenox Hill Hospital and North Shore University Hospital), Columbia University Medical Center and New York Genome Center, sharing tumor and blood samples for sequencing, relevant clinical histories, and raw sequence data (BAM files). Variant call files (VCFs) were analyzed by NY-GGC and by WGA.

### Patients

Entry criteria for this study were: minimum age of three, histologically confirmed GBM at referring institution with no requirement for central pathology review, Karnofsky score of at least 60, life expectancy of at least 6 months, and potential interest in further treatment. Clinical data was collected including results of any other clinical sequencing.

All participants provided written informed consent. Protocols were approved by local or central Institutional Review Boards at: Rockefeller University, Biomedical Research Alliance of New York (on behalf of Northwell Health), Memorial Sloan Kettering Cancer Center, New York University School of Medicine, and Weill Cornell Medicine.

### WGS and RNA-seq

Paired tumor and normal (blood) samples were sequenced from each individual and analyzed by WGS at 80X tumor and 40X normal coverage as previously described [18]. Ploidy values were used to estimate chromosome, gene, and allele copy number. We analyzed TERT promoter variants, intronic splice site variants (annotated by SnpEff), and exonic variants. Single nucleotide variants (SNVs) were classified by Tiers. Tier 1 variants are defined as variants with known clinical significance in GBM as defined by CIViC (v.alpha, 3/2015). Tier 2 variants are known to be clinically significant in another tumor type as defined by CIViC. Tier 3 are variants of unknown significance (VUS) in known actionable cancer genes with associated drugs. Tier 4 are VUS mutations in Cosmic Cancer Census Genes (v.75) [18].

Where RNA was available, RNA-seq was performed as previously described, [18] with the addition that when the RNA integrity number (RIN) score [19] was less than 7, we used the KAPA Stranded RNA-seq with RiboErase (P/N: KK8483) with Agilent SureSelectXT v6 + Cosmic (P/N: 5190–9308). We used RNA-seq data to annotate variants discovered in WGS according to the level of expression of each variant, estimated by identifying the number of supporting reads and allelic fraction. Gene expression in the tumor samples was assessed and compared with 169 GBM samples from TCGA and a modified z-score was calculated as previously described [18]. Modified z-scores of RNA-seq normalized expression data per gene was used as proxy for differential gene expression. Modified z-score per gene was calculated by subtracting the median transcripts per million (TPM) value (over the TCGA GBM cohort) from each sample’s TPM and dividing by the TCGA median absolute deviation. The z-score therefore represents the number of standard deviations each sample is from the median expression value of a specific gene of the TCGA GBM cancer cohort. For tumor-normal comparisons of splice site variants, percent spliced in (PSI) was calculated as the number of reads supporting the unannotated alternative splicing event divided by the number of canonical reads supporting the annotated event [20] and fusion transcript discovery was performed using RNA-seq data and FusionCatcher [21] as previously described [9].

When associating variants with potential therapies, we prioritized variants of high copy number focal gains and two copy homozygous loss over lower copy number whole arm gains or heterozygous losses. However, at times, lower copy number changes (< 5 copy gains or heterozygous losses) were also reported as others have done [22].

### Comparison with Watson Genomic Analytics (WGA) and targeted tumor panels

WGA is an IBM Research proof-of-concept environment of Watson for Genomics described previously, [18, 23, 24] with the addition of algorithmic updates that include basic processing of structural variants (SVs) (version 11/2016). WGA is a cloud-based cognitive system capable of analyzing mutation, gene expression, copy number alteration (CNA) and SV data provided as VCFs by leveraging 20+ structured and unstructured data sources. WGA first performs a Molecular Profile Analysis (MPA) to identify possible driver mutations and drug response biomarkers in a disease-specific manner. MPA evaluates mutations using data from structured databases such as COSMIC and ExAC to search for known variants and remove possible benign germline mutations. It also uses evidence extracted from literature using both machine-based and expert-based manual curation. These data sources are used to create a system capable of categorizing alterations as pathogenic, benign, or VUS in a disease specific manner. WGA performs a similar analysis for CNAs and gene expression changes and takes into the account the functional annotation of a gene when assessing the relevance of a CNA or differential expression. WGA also processes SVs using DNA breakpoints data output by the structural event detection program Delly [25].

From the MPA results, pathogenic and likely pathogenic alterations are assessed for potential direct and indirect (i.e. via pathway mechanisms) therapeutic options. WGA’s pathway and drug analysis identifies which therapies are most applicable and categorizes therapies by different levels of evidence from strongest, which includes FDA-recognized marker or mutations predictive of response, to the weakest, which can represent a normally appropriate therapy with a clinically supported resistance marker present in the patient. For each potential treatment option that WGA identifies, it provides all supporting evidence for the use of that therapy, mechanism of action and information on eligible clinical trials if available. WGA currently limits available treatments to molecularly targeted therapies.

To access unstructured data, particularly from literature, clinical trial information and drug label data, WGA applies Natural Language Processing (NLP). In brief, NLP requires a training phase on a high-quality corpus of papers and text that have been manually annotated by subject matter experts for terms and relationships relevant for WGA to understand, including genes, proteins, mutations, drugs, and effect. After this training phase, the NLP model is applied to the full unstructured data set, and after validation by automated methods and subject matter experts, the information is integrated into WGA.

NY-GGC provided VCFs, including CNV and gene expression files as input to WGA. Over the course of this study, IBM and NY-GGC periodically reviewed results and discussed differences between NY-GGC and WGA. Insights and feedback from these sessions along with similar sessions for other studies [24] were used to improve the process of automated curation, for example by identifying additional data sources for consideration and adjusting parameter weights. As new GBM samples became available, the entire cohort to that date was reanalyzed by the then current version of WGA.

Using NY-GGC calls as a truth set, we compared mean sensitivity and positive predictive value (PPV) across patients to determine similarity between manual and automated systems for reported variants and drug targets based on VCFs.

### Comparison with targeted panels

Prior targeted NGS panel testing results were available for 20 patients and were compared with WGS results. The percentage of variants that were in common, that were uniquely called by WGS, or that were uniquely called by the targeted panel were calculated and shown in a heatmap. Targeted NGS panel testing was done by Memorial Sloan Kettering Cancer Center IMPACT (Panel 1), FoundationOne (Panel 2), New York University Next Generation Sequencing Tumor 50 Panel (Panel 3), Weill Cornell Medical Center Precision Medicine’s whole exome sequencing assay (Panel 4), University of San Francisco’s 500 Cancer Gene Panel (Panel 5) and Caris Molecular Intel (Panel 6).

### Therapeutic targets and drug recommendations

After annotating the tumor-specific gene variants, relative to normal germline DNA, based on SNV, CNV, SV, and RNA-seq data, [18] variants were associated with drugs in the NYGC database.

NYGC database was assembled by manual curation of publically available data from the National Comprehensive Cancer Network, (https://www.nccn.org/), US Food and Drug Administration (https://www.fda.gov/Drugs/InformationOnDrugs/ApprovedDrugs), CIViC - Clinical Interpretations of Variants in Cancer (civic.genome.wustl.edu), Precision Cancer Therapy-MD Anderson (https://pct.mdanderson.org/), OncoKB (oncokb.org), canSar (https://cansar.icr.ac.uk), Pharmacogenomics Knowledgebase - PharmGKB (www.pharmgkb.org), Clinical Trials.gov (clinicaltrials.gov) and from directed literature searches. The current NYGC drug to gene database contains 260 genes associated with a least one drug.

Prioritization and rationale of drug recommendations was based on further manual assessment by the NY-GGC including but not limited to: variant Tier, quality of data supporting variant call, interpretation of the consequences of VUS in light of literature research (structural and functional analysis of protein interactions, prior knowledge of analogous variants), including analysis of X-Ray crystallographic structures, drug FDA approval status, drug identification in a current GBM trial, and record of drug success in the treatment of GBM and/or other cancer types specific to the variant.

Each individual’s results from NY-GGC were first discussed in an internal tumor board comprised of those involved in development of the analytic pipeline, bioinformaticians, project managers, pathway analysts, and clinical experts, and subsequently at a NY-GGC tumor board with that same team together with referring physicians and collaborating physicians and scientists. Further clinical use of the results provided were at the sole discretion of the referring physician.

### Statistical analysis

Mean tumor purity and ploidy were calculated with standard deviations (SD). Median number of SNVs are reported with interquartile range (IQR). Correlation between RNA-seq and exonic SNV variant allele frequency (VAF) was assessed using Pearson’s correlation coefficient. Mean number of alternative splice variants was calculated with SD. Mean sensitivity and PPV were calculated to compare agreement between the number of calls made between WGS and WGA.

## Results

### Patients and study process

Between March 2015 and July 2016, 36 patients were screened and 30 were enrolled (Table 1). Four were excluded due to final pathology indicating diagnosis other than GBM and 2 others died before sequencing began. Three participants had two separate tumor samples sequenced; one from tumors resected from two distinct brain areas, another from an enhancing and non-enhancing region on MRI, and a third from samples representing different histological characteristics. Throughout the study, we refined our processes for sample collection, squencing, analysis, interpretation, and dissemination of information to referring physicians. The average time from sample receipt to the tumor board meetings was 4.5 months (SD = 2.1) and the average time from post bioinformatic pipeline analysis to the tumor board meetings was 1.9 months (SD = 1.1).

**Table 1:** Patient characteristics

	*n* = 30
Age, median (range)	63 (25–81)
Female, no. (%)	12 (40%)
Resections, no. (%)	
Initial	19 (63%)
Biopsy only	2 (7%)
Re-resection	7 (23%)
Re-resection of subtotal	2 (7%)
Prior treatment of 7 recurrent tumors, no.	
RT/TMZ	6
Bevacizumab	3
Cetuximab	2
RT/Nivolimab	1
RT/Rindopepimut	1
CCNU	1
Gamma knife	1
Prior cranial RT, unrelated to GBM	1
Days from initial resection to sample submission, median (IQR)	67 (266)
Sample preservation, no. (%)	*n* = 33
Fresh frozen	13 (40%)
OCT embedded	12 (36%)
Formalin-fixed paraffin-embedded	8 (24%)
Tumor biomarkers, no./no. assessed	
EGFR amplification	8/20
MGMT methylation	12/30
IDH1 R132H mutation	2/30

All tumors were histologically confirmed as WHO grade IV gliomas. However, one had focal sarcomatous features, another was initially reviewed as pleomorphic xanthoastrocytoma with anaplastic features then as an epitheliod GBM upon re-revew, and a third had a PNET-like component. SD = standard deviation, IQR = interquartile range, RT = radiation therapy, TMZ = temozolomide, CCNU = Lomustine

### Tumor purity and ploidy

Tumor purity ranged from 15 to 95% (mean = 71%, SD = 16%) for all samples. Two samples had tumor purity < 20% (15 and 19%). The average estimated ploidy was 2.03 (range 1.59 to 4.06, SD = 1.235). Four samples were hyperploid and three were hypoploid.

### WGS analysis

Samples from only three patients contained a Tier 1 SNV, two with the *IDH1* R132H variant and one with *BRAF* V600E, similar to the incidence of these variants in 591 GBM patient samples in MSKCC’s cBioPortal (2 and 1% respectively). Samples from three patients contained one Tier 2 variant: *TP53* R175H, *TP53* R273C, and *PIK3CA* E542K. In contrast, there were 163 Tier 3 variants in 90 genes across 29 samples. There were 143 variants in Tier 4 genes, but none were deemed targetable. Only one sample had no SNVs in targetable genes. In total, 44 genes and 32 samples contained targetable SNVs, the vast majority of which were Tier 3 variants.

Among clinically actionable SNVs, *PIK3R1* and *RB1* were mutually exclusive, consistent with previous observations [26, 27]. Ten SNVs were identified in *PIK3CA* and *PIK3R1*. Of the three variants identified in *PIK3CA*, one was assigned to Tier 2 (E542, within the catalytic subunit), and the other two were assigned to Tier 3. Seven Tier 3 variants were identified in *PIK3R1*, all of which were in the iSH2 regulatory domain.

All but one sample had CNVs that were considered targetable. We observed 24 arm-scale and nine focal losses of chromosome 10 that included *PTEN*, consistent with previous observations in GBM [28–30]. Of those 24, we observed 10 cases containing a secondary SNV. EGFR focal amplifications were observed containing the *EGFRvIII*, *EGFRvV* and A289V variants. Six patients were previously treated with temozolomide; two had copy number losses of CDKN2A, and one also had a copy number loss of RB1 [26].

**Fig. 1:**
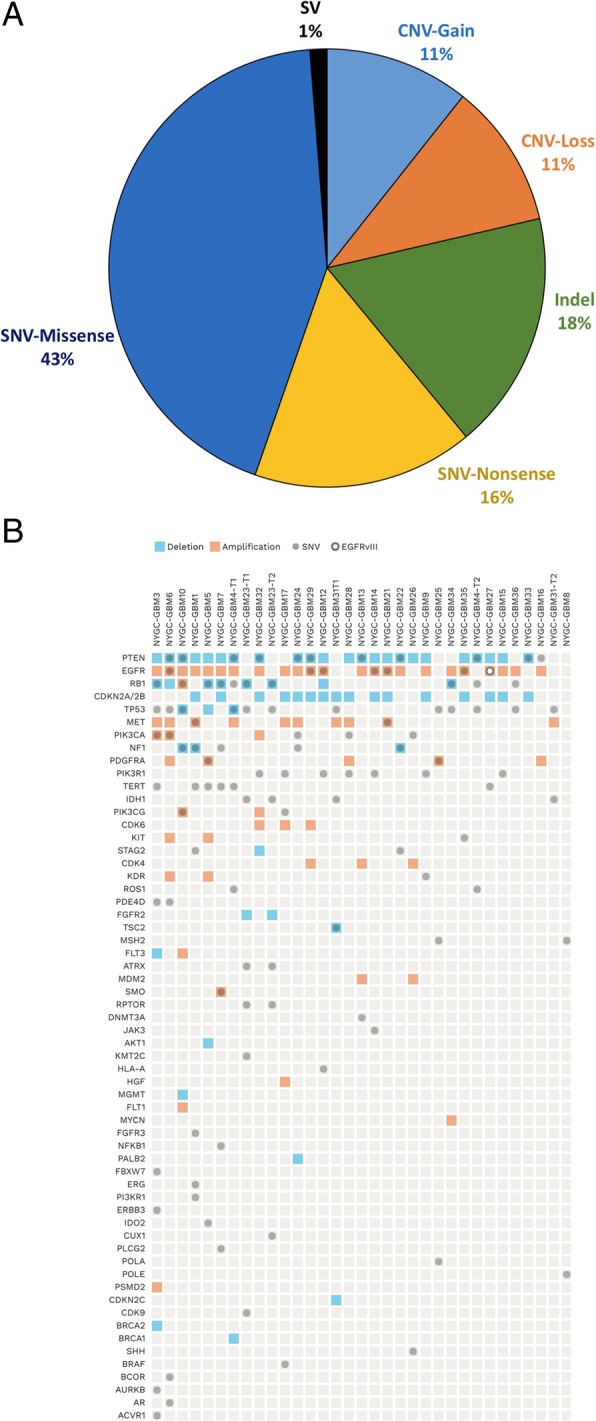
Somatic alterations associated to potential therapy in NY-GGC study. **a** Distribution of therapeutic associations with single nucleotide variants (SNV), copy number variations (CNV), insertions/deletions (Indel) and structural variations (SV) discovered from whole genome sequencing (WGS) data in the NY-GGC study 33 sample data set. **b** Matrix outlining the types of variants per gene discovered by WGS across the cohort. Blue boxes indicate copy number losses, orange boxes indicate copy number gains, grey circle represents a somatic nucleotide variant, and open circle is specific to the EGFR vIII SV.

*TERT* promoter mutations, which can have prognostic consequences depending on factors such as MGMT promoter methylation status, were found in 19 of 33 samples, consistent with prior studies. [31–34] There were 35 to 5881 (median = 157, IQR = 111) exonic SNVs per sample, of which two were exceptionally high (1954 and 5881), one previously treated with temozolomide and one not. Missense mutations accounted for 43% of calls that resulted in drug associations, and CNV and SV data were used in 23% of therapeutic associations (Fig. 1a).

### RNA analysis

**Fig. 2:**
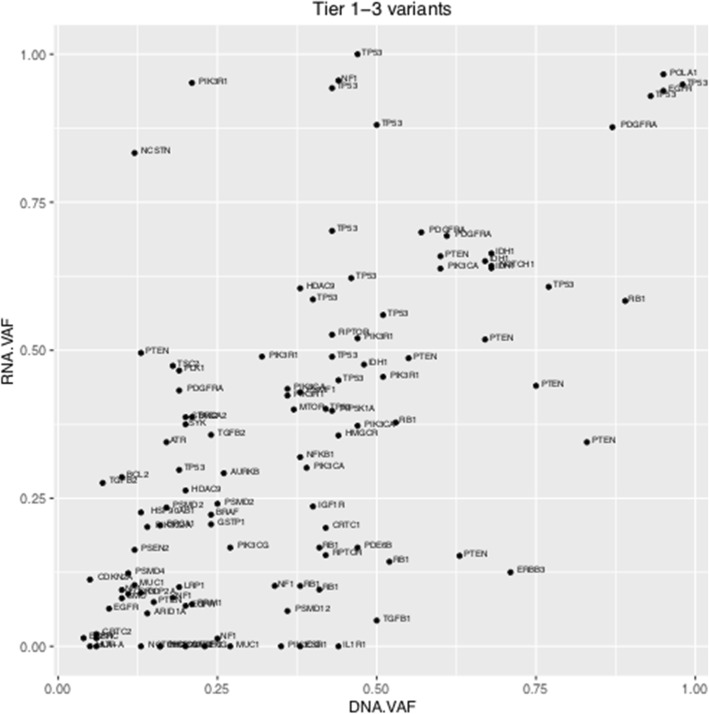
DNA variant allele frequency correlation with RNA variant allele frequency. Correlation of variant allele frequency (VAF) in WGS vs RNA-seq plotted for all Tier 1–3 variants with sufficient (≥5 total reads) coverage in RNA

Of 30 samples, sufficient RNA quantity for library preparation was extracted from 27 and high quality sequences with sufficient coverage were obtained from 26. There was good correlation in the VAF between RNA-seq and exonic SNVs identified by WGS (Pearson’s correlation coefficient r = 0.622, *p*-value = 1.899e-13, Fig. 2). RNA-seq identified 113 of 155 (73%) variants identified by WGS. We observed more similarity in allelic frequencies in DNA and RNA data for genes with higher read count in a specific sample (i.e. overamplified DNA and higher RNA transcript count).

**Fig. 3:**
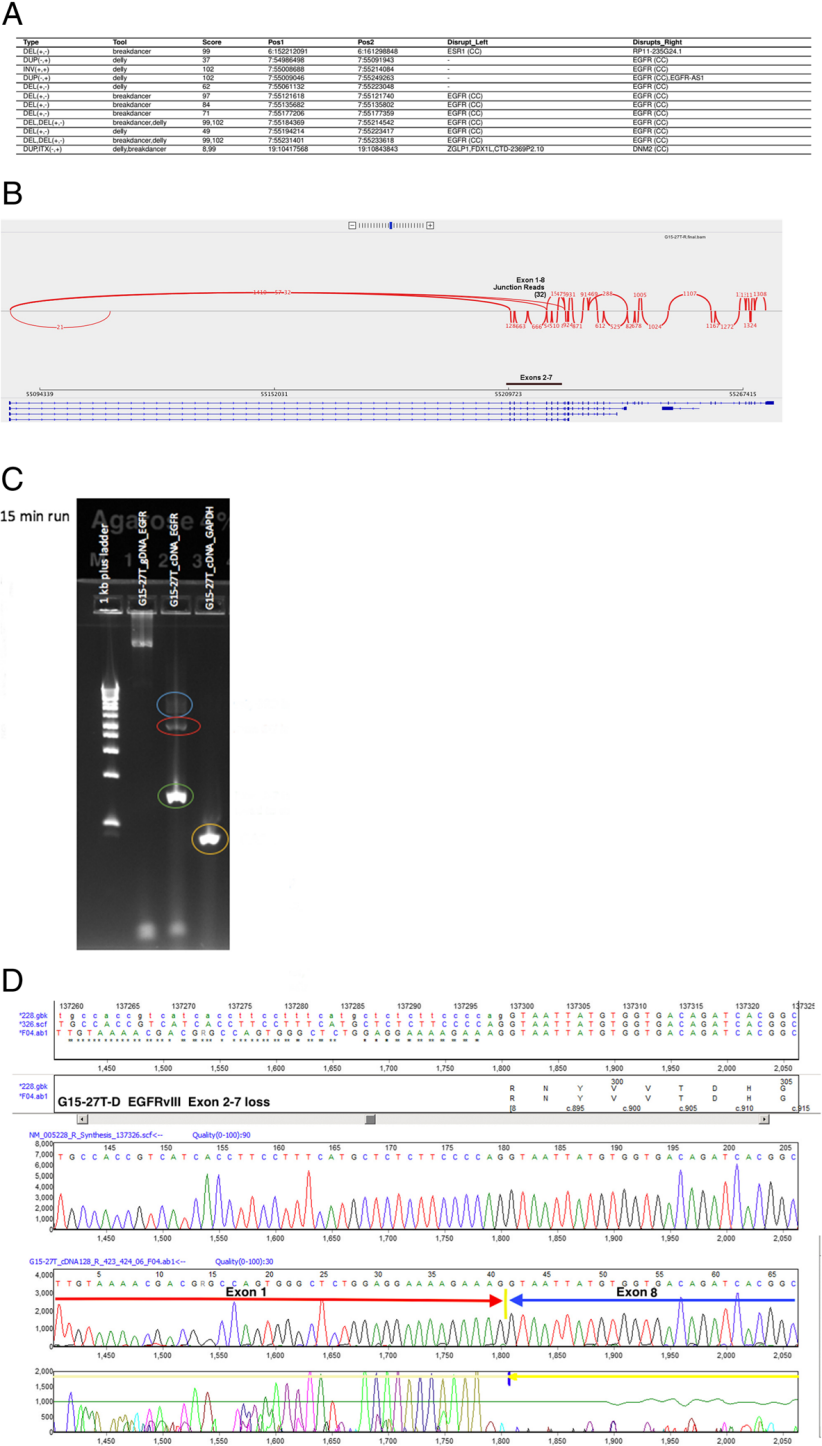
EGFR vIII detection in NYGC-GBM 27. **a** Table of EGFR SVs called by two SV callers (Delly, Breakdancer) in WGS data. **b** Sashimi plot from RNA-Seq data indicating 32 Exon1–8 junctions reads (exon 2–7 del). **c** RT-PCR confirmation of EGFRvIII. Blue circle indicates wild-type EGFR (exons 1–8) 929 bp fragment, green circle indicates EGFRvIII exons 2–7 loss 128 bp, red circle indicates EGFR potential exon 6–7 loss, yellow circle indicates GAPDH 87 bp control. **d** Sanger sequencing of gel band confirming presence of EGFR vIII with Glycine codon insertion as previously reported, (Chr7: 55,087,058-55,223–523; c.335_1135delinsggt, p.V30_R297delinsG)

**Fig. 4:**
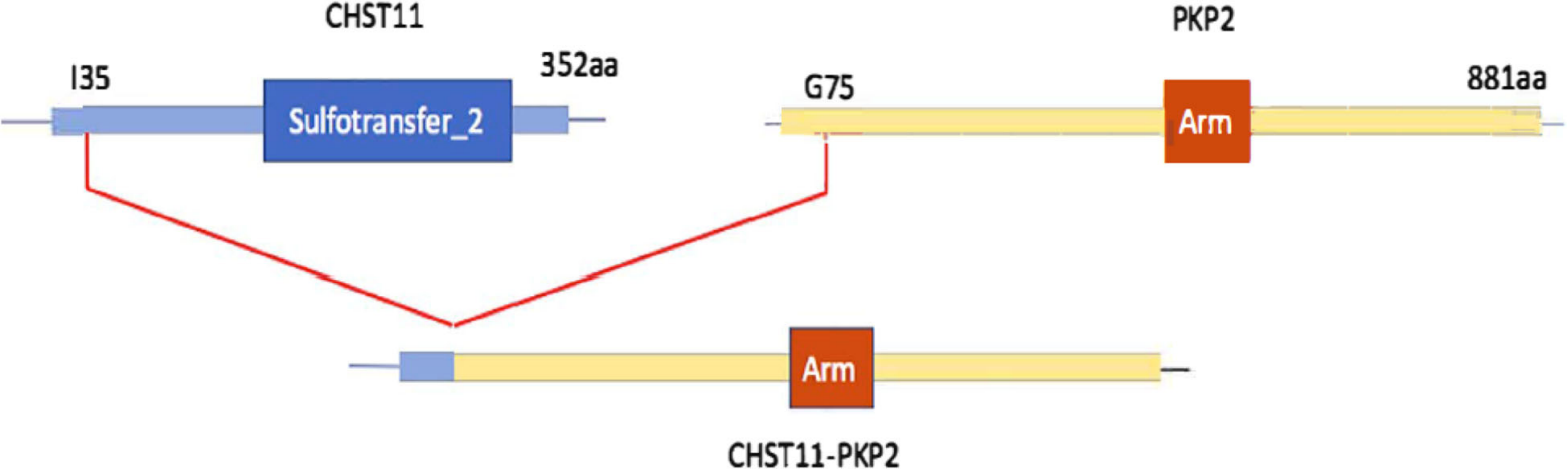
CHST11-PKP2 fusion in NYGC-GBM13. Fusion transcript between the amino-acids 1 to 35 of CHST11 and amino-acids 75 to 881 of PKP2. Breakpoints are: CHST11: Chr12:104851292:+ and PKP2: Chr12:33031966:-.The fusion is predicted to be in-frame and PKP2’s Armadillo domain (Arm) is preserved

RNA-seq revealed an average of seven alternative splice variants (PSI > 10%) in Cancer Census genes per individual (SD = 5.02, range 1 to 26). One variant (c.2419-274_2443del) in NYGC-GBM1 disrupted a splicing acceptor site and generated an exon 11 skipping event in the MET transcript. [18] Additionally, both *EGFR*vIII and *EGFR*vV exon deletions were confirmed in RNA-seq data (Fig. 3). We also discovered a *CHST11-PKP2* fusion (NYGC-GBM13, Fig. 4). One of the breakpoints disrupted *CHST11* and the other was in the intergenic region just upstream of *PKP2*. The *CHST11* promoter may drive a high expression of this fusion transcript (modified z-score of RNA-seq normalized expression =3.01). *PKP2* is associated with *EGFR* regulation and this fusion may result in activation of *EGFR* signaling pathway [35].

### Identification and utilization of potentially clinically actionable information

We identified one or more potential treatment targets in all 30 tumor samples. WGS/RNA-seq identified 61 disrupted genes, including both SNVs and CNVs, which were associated with 87 targeted treatment options including 62 clinical trials for 39 therapies (Table 2). The most commonly identified therapeutic targets were *PTEN, EGFR*, *CDKN2A,* and *MET* (Fig. 1b). Two samples with high mutation burden were found to have Tier 4 variants in *MSH2*, a mismatch repair (MMR) gene for which checkpoint inhibitors were identified as having potential utility to increase immune response to tumors with high neoantigen load [36].

**Table 2:** Key targetable variants and associated drugs and clinical trials

Sample	Key Therapeutic Variants	Associated Drugs	Clinical Trials
GBM1	PIK3R1 R562del, MET R755fs MET 11, focal gain	BKM120, INC280	NCT01870726 NCT02386826
GBM3	PIK3CA V344G	BKM120	NCT01870726 NCT01349660
GBM4	EGFR gain, PTEN W111C, whole arm loss	Cetuximab, Everolimus	NCT01238237 NCT01870726
GBM5	PDGFRA/KIT/KDR gain	Nilotinib	NCT01140568 NCT01871311
GBM6	PIK3CA G542K, PTEN N59 fs, EGFR whole arm gain	BKM120, Everolimus, Cetuximab	NCT02142803 NCT01349660 NCT01238237
GBM7	SMO R421*, NF1 Q270*	Vismodegib, MEK162	NCT00980343 NCT01885195
GBM8*	POLE P1505S, MSH2 splice site donor c.366 + 1G > A, High mutation burden	Pembrolizumab, Nivolumab	NCT02337686 NCT02017717
GBM9	EGFR gain, KDR R1022*, PIK3R1 E443del	Cetuximab, ABT41, Bevacizumab, BKM120	NCT02573324 NCT01349660
GBM10	MGMT loss, PTEN R130*, whole arm loss NF1 E1722*	Temozolomide, Everolimus, MEK162	NCT01885195
GBM12	EGFR A289V, focal gain, PIK3R1 D560G	BKM120, Afatanib	NCT01349660 NCT01934361
GBM13	MDM2 focal gain, CDK4 focal gain, PTEN R47S	RG7112/RG7388, AMG232, Palbociclib, Everolimus	NCT01877382 NCT02143635 NCT01227434 NCT01870726
GBM14	EGFR A289V, focal gain, PIK3R1 W597G, PTEN whole arm loss and focal deletion	BKM-120, Everolimus	NCT01349660 NCT01934361 NCT01870726
GBM15	PIK3R1 T473P, EGFR whole arm gain, CDKN2A homozygous focal deletion, PTEN whole arm loss	BKM-120, Cetuximab, ABT-414, Afatanib, ABBV-221, Palbociclib, Ribociclib, Everolimus	NCT01339052 NCT02423525 NCT02573324 NCT02365662 NCT01227434 NCT02345824
GBM16	PTEN Y16*, EGFR focal gain, PDGFRA focal gain	Everolimus, Cetuximab, ABT-414, Afatanib, ABBV-221, Nilotinib, Crenolanib	NCT02423525 NCT02573324 NCT02365662 NCT02626364 NCT01140568 NCT01871311
GBM17	BRAF V600E, EGFR gain	Vemurafanib, Cobimetinib, ABT414	NCT02537600 NCT02573324 NCT02423525
GBM21	EGFR R222C, focal gain, MET P791L, focal gain, PTEN whole arm loss, CDKN2A homozygous focal deletion	Cetuximab, ABT-414, Afatanib, ABBV-221 Crizotinib, INC280, Everolimus, Palbociclib, Ribociclib	NCT02423525 NCT02573324 NCT02365662 NCT02540161 NCT02034981 NCT02386826 NCT01227434
GBM22	PTEN V119F, whole arm loss, STAG2 focal deletion, NF1283fs, focal loss, TP53 R158H	Everolimus, Olaparib, Veliparib, MEK162, Temsirolimus/Docetaxel	NCT01390571 NCT02152892
GBM23	IDH1 R132H, RPTOR A578G	AG-120, AG-881, BAY-1436032, Everolimus, INK128	NCT02073994 NCT02481154 NCT02746081 NCT01434602 NCT02142803
GBM24	PIK3CA R93W, EGFR whole arm amplification, MET focal gain, PTEN R335*, T277I, whole arm loss, CDKN2A homozygous focal deletion, PALB2 whole arm loss	BKM-120, Cetuximab, ABT-414, Afatanib, ABBV-221, Crizotinib, INC280, Everolimus, Palbociclib, Ribociclib, Olaparib, Veliparib	NCT01870726 NCT01339052 NCT02423525 NCT02573324 NCT02365662 NCT02386826 NCT01227434 NCT02345824 NCT01390571 NCT02152982
GBM25	POLA1 G1178, MSH2 splice site donor c.366 + 1G > A, TP53 R175H, G245S, PDGFRA Y375H, PDGFRA/KIT/KDR focal gain, High mutation burden	Pembrolizumab, Nivolumab, Paclitaxel, Nilotinib	NCT02337686 NCT02017717 NCT02379416 NCT01140568 NCT01871311
GBM26	PIK3CA R88Q, MDM2 focal gain, CDK4 focal gain, PTEN whole arm loss	BKM-120, RG-7112, AMG-232, Palbociclib, Ribociclib, Everolimus	NCT01249660 NCT01339052 NCT01877282 NCT01723020 NCT01390571 NCT02152982 NCT02255461
GBM27	EGFRvIII, EGFR focal gain, CDKN2A homozygous focal deletion, PTEN whole arm loss	Afatnib, Rindopepimut, CAR-T, Cetuximab, ABT-414, Afatanib, Palbociclib, Ribociclib, Everolimus	NCT01480479 NCT02423525 NCT02664363 NCT02573324 NCT02423525 NCT00703625 NCT01390571 NCT02152982
GBM28	PIK3R1 Q579fs, PIK3CA D939G, MET focal gain, PDGFRA focal gain, CDKN2A homozygous focal deletion, PTEN whole arm loss	BKM-120, Crizotinib, INC280, Nilotinib Palbociclib, Ribociclib, Everolimus	NCT01870726 NCT01339052 NCT01870726 NCT01339052 NCT02365662 NCT01140568 NCT01390571 NCT02152982
GBM29	EGFR A289V, EGFR focal gain, PTEN R130*, whole arm loss, CDKN2A homozygous focal deletion, CDK4 focal gain	Cetuximab, ABT-414, Afatanib, Everolimus, Palbociclib, Ribociclib	NCT02573324 NCT02423525 NCT0070362 NCT01390571 NCT02152982 NCT01390571 NCT02152982
GBM31	IDH1 R132H, TSC2 P1215fs, TP53 R175H, CDKN2A homozygous focal deletion	AG-120, AG-881, BAY-1436032, Everolimus, Temsirolimus, MLN0128, Temsirolimus/Docetaxel, Palbociclib, Ribociclib	NCT02073994 NCT02481154 NCT02746081 NCT002238946 NCT02142803 NCT01390571 NCT02152982
GBM32	PIK3R1 A483P, STAG2 focal deletion, PTEN M198R, whole arm loss	BKM-120, Olaparib, Veliparib, Everolimus	NCT01870726 NCT01390571 NCT02152982 NCT01434602
GBM33	PTEN Q97*, whole arm loss, CDKN2A focal loss	Everolimus, Temsirolimus, Palbociclib, Ribociclib	NCT01390571 NCT02152982
GBM34	EGFR gain, PTEN whole arm loss, TP53 C242S, V143 M, MYCN gain	Cetuximab, ABT-414, Afatanib, Everolimus, Temsirolimus, Docetaxel, CP-0610, MK-8628, GSK2820151	NCT02573324 NCT02423525 NCT00703625 NCT02698176 NCT02630251 NCT01877382
GBM35	EGFR focal gain, PIK3R1 L372dup, CDKN2A homozygous loss, PTEN whole arm loss, KIT A207V	Cetuximab, ABT-414, Afatanib, BKM-120 Palbociclib, Ribociclib, Everolimus, Imatinib, Nilotinib	NCT02573324 NCT02423525 NCT02345824 NCT01390571 NCT02152982
GBM36	NF1 c.1062 + 1 Splice Site Donor, TP53 T211I	MEK162, Temsirolimus, Docetaxel	NCT00703625

* Final therapeutic association performed post-mortem

We identified potentially synergistic combination therapy options such as those targeting multiple mutations occurring in the same pathway or targeting multiple arms of the same pathway in seven patients. Three variant types, *EGFR* amplification, *PTEN* loss, and PIK3R1 SNVs were most frequently associated with potential combination targets. In NYGC-GBM24, *EGFR* gain and *PTEN* loss were identified, which could independently activate the PI3K/AKT pathway. Cetuximab and everolimus were suggested to inhibit activation of EGFR and mTOR downstream of AKT, respectively [37]. In NYGC-GBM1, *PTEN* loss, *MET* gain and *PI3KR1* variants were present, and associated with a PI3K inhibitor (BKM120) and a MET inhibitor (INC280). [18, 38, 39] NYGC-GBM9 had a PIK3R1 variant and *PTEN* loss and a PI3K inhibitor (BKM120) and everolimus were suggested [40]. In NYGC-GBM17 with *BRAF* V600E, *PIK3R1* loss, and *MET* and *EGFR* gain, the combination of vemurafenib and INC280 were identified, aiming to avoid resistance to inhibitors targeting *BRAF* V600E [41–44].

We considered levels of expression in pathways downstream of potentially targetable variants. NYGC-GBM7 contained an *EGFR* gain and a nonsense SNV in the gene Smoothened (*SMO*), which is directly targetable by vismodegib. The *SMO* variant (p.Arg421*/c.1261C > T) is of unknown function. It was confirmed by RNA-seq and Sanger sequencing, however no significant alteration in *SMO* transcript level (z-score = − 0.93) was identified. SMO activation leads to release of GLI1 from SUFU-mediated cytoplasmic sequestration and to nuclear translocation and transcription activation [45]. Nonsense mutations are often not directly associated with RNA decay, however RNA-seq analysis showed significant upregulation of downstream transcripts in the SMO-SUFU-GLI pathway, including those encoding *MDM2* and *IGFBP6,* which are associated with tumor proliferation [46]. Vismodegib, was well tolerated, but not efficacious in recurrent GBM [47] but given that this study did not stratify enrollees based on genomic variants, it was considered potential therapeutic option.

Of 30, there were four patients in whom the WGS/RNA-seq done in this study were used to make treatment decisions. NYGC-GBM17 was a 33 year-old female whose initial pathology was reviewed as a low-grade pleimorphic xanthroastrocytoma (PXA), later re-reviewed as an epitheliod glioblastoma. She was treated with radiation, temozolomide, and multiple procedures for cyst drainage. NY-GGC identified a *BRAF* V600E variant, also found by FoundationOne, which has been reported to occur in both PXA and epitheliod GBM tumors, and this finding supported a recent case report suggesting that epithelioid GBM may arise from a PXA with a *BRAF* V600E mutation [48]. After presentation at the NY-GCC tumor board, the patient was treated with drugs targeting BRAF (dabrafenib and trametinib) for 12 months. At the time of this writing, she was 18 months post initial diagnosis, free of focal neurologic deficits with stable disease on MRI.

NYGC-GBM25 was a 24 year-old man with a partially resected GBM that recurred and was re-resected 1 month later. He was treated with chemoradiation and intra-arterial Bevacizumab on a clinical trial (NCT 01811498). NY-GGC identified a high mutational burden, which was likely due to a truncating mutation in a MMR gene, *MSH2* [49–51] and a missense VUS in POLA1, which combined with MMR defects can lead to higher rates of mutation than MMR defects alone [52]. At NY-GGC tumor board, checkpoint inhibitors were identified as possible targeted treatments, based on evidence that they are effective against MMR-defective tumors and that there may be potential benefit of concurrent therapy [53, 54]. The referring physician stopped adjuvant temozolomide, in light of evidence that MSH6 protein loss could lead to progression on temozolomide in GBM, [55, 56] and started nivolumab. Shortly thereafter, the patient underwent re-resection and this sample was submitted for resequencing in another study. This sample, collected 7 months after the initial sample, had a similarly high mutation burden with many of the same potentially targetable variants. The hypermutation phenotype was again chosen by the physician as the focus of treatment, discontinuing nivolumab and starting pembrolizumab. The patient later suffered multiple central nervous system infections and seizures and died 6 months after the third resection.

NYGC-GBM12 was a 64 year-old male treated with temozolomide, bevacizumab, intra-arterial cetuximab (based on FISH evidence of EGFR amplification) and multiple resections. NY-GGC identified an extreme focal EGFR amplification, and suggested erlotinib despite pre-treatment with cetuximab. He received 3 cycles of erlotinib but did not have a significant clinical response and died 5 months later.

Overall, NY-GGC tumor boards occured at an average of 4.5 months (SD = 2.1 months) after sample submission and follow up indicated that results were utilized in the care of at least three patients at the time of this writing. At least eight patients died or experienced significant functional decline before their physicians received NY-GGC results.

### Concordance of WGS/RNA-seq and panel-based diagnostic reports

**Fig. 5:**
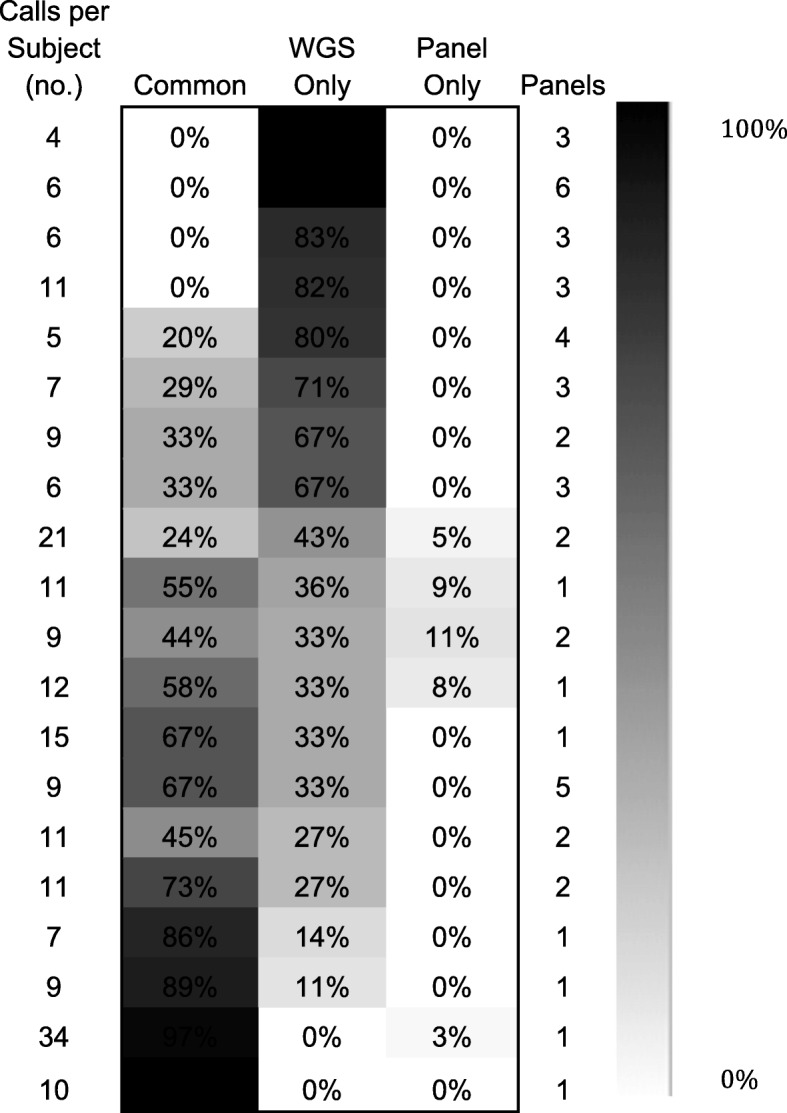
WGS vs focused NGS panel comparison. Number of calls made based on WGS versus calls made by focused NGS panel testing per patient. Blue indicates variant calls made uniquely by NY-GGC, green indicates variant calls made uniquely by panel testing, yellow indicates common calls made, purple indicates germline variants called as tumor variants by panel testing; *panel testing done on subsequent sample, **panel testing done on prior sample, ^partial report of panel testing available, Panel 1 = Memorial Sloan Kettering Cancer Center IMPACT Panel, Panel 2 = FoundationOne, Panel 3 = NYU Next Generation Tumor 50 Panel, Panel 4 = Weill Cornell Institute of Precision Medicine’s whole exome sequencing assay, Panel 5 = University of California San Francisco’s 500 Cancer Gene Panel, Panel 6 = Caris Molecular Intelligence’s profile which includes next generation sequencing analysis of 44 genes as well as other assays such as immunohistochemistry

Twenty of the 30 GBM patients had targeted panel-based sequencing performed (Fig. 5). Significantly, we identified targetable variants not identified by panels in 18 of 20 (90%) cases**.** There were a median of 4 (IQR = 2) WGS/RNA-seq unique treatment targets, 5 (IQR = 5.5) calls in common, and 0 (IQR = 0.25) panel-unique calls per case. The number of common calls varied by panel type; WGS/RNA-seq and Panel 1, which incorporates a matched normal, had 82% of calls in common. Thirteen percent of calls were in common with Panel 3, which does not include a matched normal; we identified germline variants falsely described as tumor variants by the panel in two of five individuals. Panel 2, which also does not sequence a matched normal, reported a total of 10 tumor variants across five reports (providing therapeutic implications for one) which WGS/RNA-seq identified as germline variants. In four of the 20 cases, the panel identified one unique variant that WGS/RNA-seq did not, largely due to low VAF.

In sum, 84 additional clinically actionable calls were made using WGS/RNA-seq that were not identified by panels, compared to four made by panels and missed by WGS/RNA-seq. Of all 200 potentially actionable variants identified, panels did not identify 39.5%, and WGS/RNA-seq did not identify 2.5%. Out of the 44 calls made by two panels without matched normals, 13 (30%) were germline.

### Concordance with WGA

**Fig. 6:**
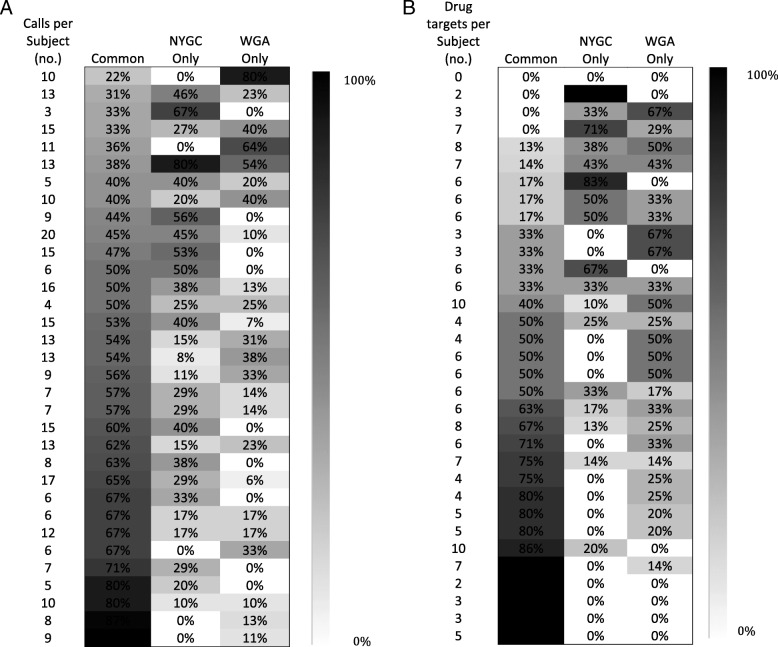
Expert manual versus automated treatment target curation comparison. **a** Variants identified by expert manual versus automated treatment target curation. **b** Drug targets identified by NY-GGC’s expert manual versus WGA’s automated processing when the same variants were identified

WGA achieved good agreement with NY-GGC when comparing the reported variants (mean sensitivity = 0.71, SD = 0.18/PPV = 0.880, SD = 0.20 across patients, Fig. 6a). WGA and NY-GGC maintained different thresholds for identifying certain types of variants. For example, NY-GGC often identified genes with copy number gains of less than five and heterozygous losses as potentially targetable while WGA did not. NY-GGC normalized some CNV calls, for example to account for tumor purity. NY-GGC also manually reviewed known cancer genes to identify SNVs that were below threshold (15% VAF) and had at least 40 total read count. Moreover, at the time of initial analysis, WGA was just beginning to be trained to identify SVs as targetable, whereas NY-GGC comprehensively noted SVs and associated therapies.

There was also good agreement in the drugs recommended when the same variants were identified and when considering similarly scoped therapies (mean sensitivity = 0.74, SD = 0.34/PPV = 0.79, SD = 0.23 across patients, Fig. 6b). As an example of one discrepancy, in NYGC-GBM13, NY-GGC prioritized *MDM2* amplification as a potential targetable variant, while WGA selected *CDK4* amplification. WGA did not associate *MDM2* with an inhibitor because open trials were not for patients with GBM. NY-GGC prioritized *MDM2* over *CDK4* therapy based on in vitro studies that *CDK4* amplification alone is not sufficient for CDK4 inhibitor sensitivity in cell lines. [57] The CDK4 inhibitor, palbociclib, also had contradicting evidence regarding blood-brain barrier penetration, [58] and tumor board discussions of preliminary data of the GBM clinical trial of palbociclib, later terminated, revealed that the physicians were less likely to recommend this drug. WGA focused on reporting only molecularly targeted therapies; thus chemotherapies and immunotherapies were outside its scope. WGA also did not offer therapeutic options for VUS where NY-GGC did, based on literature suggesting potential oncogenicity and targetability. Finally, in creating drug databases, WGA used an automated, comprehensive, and unbiased approach that included scanning PubMed abstracts with minimal manual filtering and incorporated publically available drug information resources, while NY-GGC’s was created by manual review of the literature and refined through tumor boards and interdisciplinary discussions often including unpublished data. Therefore, NY-GGC’s therapeutic associations adapted with knowledge and insight gained throughout the study.

## Discussion

We undertook a comparative genomic-based study of GBM patients to investigate the utility of targeted panels versus WGS/RNA-seq and evaluated the feasibility and reliability of results obtained with manual versus automated curation by IBM’s WGA. Together, WGS/RNA-seq was more sensitive than panels, detecting 39.5% more calls than panels, and 97.5% of the calls found by panels. Despite these findings, potentially clinically actionable calls, made in 100% of cases, altered treatment plans in only 10% of cases. This is likely a consequence of a number of factors, including availability of drug and concern of side effect profiles, lack of familiarity and slow acceptance of new technology, appropriate timing of sequencing within the patient’s clinical course, lack of prior clinical information to rigorously assess risk-vs-benefits concerns, and clinician perception of the usefulness and relevance of the sequencing data based on time elapsed between resection and sample submission; further research is warranted to identify the biggest barriers to implementation and ways to overcome them.

The majority of potentially therapeutic associations were identified in targetable genes (Tier 3) but with a variant unknown to be actionable. Such variants were prioritized when there was evidence that they would likely affect protein function, similarly to Tier 1–2 variants. These findings were typically complex and required manual curation of information from the literature, necessitating many person-hours per case. [18] The interpretation of splicing variants and structural variants resulting in splicing aberrations, only detected by combining WGS [59] and RNA-Seq remains challenging, especially in the context of individual sample or small cohort analyses. There may also be additional connections between variants and pathways that we did not identify. Furthermore, the therapeutic associations made are based on what was available in the literature and in clinical trials at the time of analysis and interpretation of that individual sample and will evolve with new drug development.

Hence a critical component of improving scalability of data interpretation will be with automation. Here we explored this approach through a comparative analysis of manual expert with automated curation. The time required for WGA to match VCF calls with drug options improved to within 6 min over the course of the study, while manual expert curation was an average of 1.9 months. While WGA requires further development, for example, to consider SVs as targets routinely or to expand the drug database beyond molecularly targeted therapies, the potential usefulness of this timeframe in the clinical setting is clear.

The per-patient cost of targerted panels at cancer centers are generally about $1000 and FoundationOne is currently ~$5800 [60]. By comparison, the all-in cost of clinical WGS/RNAseq and analysis by all platforms in the current study was ~$10,000. While financial cost-benefit analysis may currently favor panels or WES, our results suggest an imminent future in which the technical advantages and breadth of WGS/RNA-seq will increasingly provide improved cost-benefits by returning the most comprehensive analysis of tumor mutations. As costs and efficiencies improve, it may become reasonable to consider how to routinely apply WGS/RNA-seq to benefit cancer patients.

In addition to lowered cost and timely interpretation of sequencing data, other strategies towards improved implementation of WGS/RNA-seq include submission of samples as soon as possible after resection so that results are available for consideration at the time treatment decisions are made. This requires both clinician education about the utility of such an assay as well as the availability of clinically approved tests. NYGC has recently obtained such regulatory approval from the New York State Department of Health. One of the biggest challenges in doing so in WGS was in demonstrating reproducibility at far less depth (80X/40X for tumor normal pair) than depths of 100-200X for exomes and 500X for panels. Specifically, true negatives remain elusive even with very high depths because oncology variant callers look for variants and not for correct base calls, and remain a limit of this assay. It will be interesting to see how this influences adoption of WGS/RNA-seq for somatic variants in the coming months.

Limitations of this study include the inclusion of multiple sample preservation methods. This may have affected the variant calls, although previous studies have shown that 98% of actionable calls made in fresh frozen samples can also be made in FFPE samples. [61] Although actionable calls were made for all samples, some calls may have been missed in those samples that had tumor purity of less than 20%. [62] Another limitation is the small sample size. This sample may not have been representative of other patients with GBM. Furthermore, only 20 of the 30 patients enrolled had prior targeted panel with which the WGS analysis could be compared. To address this, we are conducting a follow-up study of 200 patients.

## Conclusion

In sum, when we compared manual and automated searches for therapeutic options, WGA offered a broader array of options with a much faster processing time while maintaining significant sensitivity. Meanwhile, NY-GGC’s manual curation associated clinical significance to a broader array of variants, suggested combination therapies, and incorporated feedback regarding individual patient clinical data as well as physician concerns discussed at tumor board meetings. Taken together, this study points to the potential of WGS/RNA-seq, combined with automated curation, to maximize therapeutic options which can be used in clinical decision making for the benefit of cancer patients.

## Abbreviations

CNV: copy number variant
GBM: glioblastoma
IQR: interquartile range
MMR: mis-match repair
MPA: molecular profile analysis
NLP: natural language processing
NYGC: New York Genome Center
NY-GGC: New York Glioblastoma Genome Consortium
PPV: positive predictive value
PSI: percent spliced in
PXA: pleiomorphic xanthroastrocytoma
RIN: RNA integrity number
RNA-seq: RNA sequencing
SD: standard deviation
SNV: single nucleotide variant
SV: structural variant
TCGA: The Cancer Genome Atlas
TPM: transcript per million
VAF: variant allele frequency
VCF: variant call file
VUS: variant of unknown significance
WES: whole exome sequencing
WGA: Watson Genomic Analysis
WGS: whole genome sequencing
