# Correction to: Sequencing and curation strategies for identifying candidate glioblastoma treatments

**DOI:** 10.1186/s12920-019-0563-y

**Published:** 2019-08-02

**Authors:** Mayu O. Frank, Takahiko Koyama, Kahn Rhrissorrakrai, Nicolas Robine, Filippo Utro, Anne-Katrin Emde, Bo-Juen Chen, Kanika Arora, Minita Shah, Heather Geiger, Vanessa Felice, Esra Dikoglu, Sadia Rahman, Xiaolan Fang, Vladimir Vacic, Ewa A. Bergmann, Julia L. Moore Vogel, Catherine Reeves, Depinder Khaira, Anthony Calabro, Duyang Kim, Michelle F. Lamendola-Essel, Cecilia Esteves, Phaedra Agius, Christian Stolte, John Boockvar, Alexis Demopoulos, Dimitris G. Placantonakis, John G. Golfinos, Cameron Brennan, Jeffrey Bruce, Andrew B. Lassman, Peter Canoll, Christian Grommes, Mariza Daras, Eli Diamond, Antonio Omuro, Elena Pentsova, Dana E. Orange, Stephen J. Harvey, Jerome B. Posner, Vanessa V. Michelini, Vaidehi Jobanputra, Michael C. Zody, John Kelly, Laxmi Parida, Kazimierz O. Wrzeszczynski, Ajay K. Royyuru, Robert B. Darnell

**Affiliations:** 1 New York Genome Center, 101 Avenue of the Americas, New York, NY 10013 USA; 2 Laboratory of Molecular Neuro-Oncology, The Rockefeller University, 1230 York Avenue, New York, NY 10065 USA; 3 IBM Thomas J. Watson Research Center, Yorktown Heights, NY 10598 USA; 4 Northwell Health, Lenox Hill Hospital, 100 E. 77th Street, New York, NY 10075 USA; 5 Northwell Health, The Brain Tumor Center, 450 Lakeville Road, Lake Success, Lakeville, NY 11042 USA; 6 New York University, School of Medicine, 550 First Avenue, New York, NY 10016 USA; 7 Memorial Sloan-Kettering Cancer Center, 1275 York Avenue, New York, NY 10065 USA; 8 Columbia University Medical Center, 710 West 168th Street, New York, NY 10032 USA; 9 Hospital for Special Surgery, 535 E. 70th Street, New York, NY 10021 USA; 10 IBM Watson Health, NW Broken Sound Bkwy, Boca Raton, FL 33487 USA; 11 Howard Hughes Medical Institute, The Rockefeller University, 1230 York Avenue, New York, NY 10065 USA; 12 Present address: Google, 76 9th Avenue, New York, NY 10011 USA; 13 Present address: Rockefeller University, 1230 York Avenue, New York, NY 10065 USA; 14 Present address: 23&Me, 899 W Evelyn Ave, Mountain View, CA 94041 USA; 15 Present address: Max Planck Institute of Immunobiology and Epigenetics, Stübeweg 51, D-79108 Freiburg, Germany; 16 Present address: The Scripps Research Institute, 10550 N. Torrey Pines Road, La Jolla, CA 92037 USA; 17 Present address: The Tisch Cancer Institute, 1470 Madison Avenue, New York, NY 10029 USA; 18 Present address: Memorial Sloan-Kettering Cancer Center, 1275 York Avenue, New York, NY 10065 USA; 19 Present address: Harvard Medical School, 10 Shattuck Street, Boston, MA 02115 USA; 20 Present address: Yale School of Medicine, 333 Cedar Street, New Haven, CT 06510 USA

**Frank et al. BMC Med Genomics**

**https://doi.org/10.1186/s12920-019-0500-0**

Following publication of the original article [1], it was reported that the given name of the fourteenth author was incorrectly published. The incorrect and the correct names are given below.

Incorrect: Alice Fang

Correct: Xiaolan Fang
